# Role of Gelatinases MMP-2 and MMP-9 in Healthy and Complicated Pregnancy and Their Future Potential as Preeclampsia Biomarkers

**DOI:** 10.3390/diagnostics11030480

**Published:** 2021-03-09

**Authors:** Asparuh Nikolov, Nikola Popovski

**Affiliations:** 1 Cardiovascular Research Working Group, Division of Medicine, Institute for Scientific Research, Medical University-Pleven, 5800 Pleven, Bulgaria; 2 Clinic of Obstetrics and Gynaecology, Department of Obstetrics and Gynaecology, University Hospital Pleven, Medical University-Pleven, 5800 Pleven, Bulgaria; n_popovsky@abv.bg

**Keywords:** extracellular matrix, spiral arteries, collagen types I and IV, microcirculatory ischemia, biomarkers, preeclampsia

## Abstract

Gelatinases (matrix metalloproteinase-2 and -9) are enzymes from the matrix metalloproteinases (MMPs) family, which are associated with collagen degradation. MMP-2 is capable of cleaving gelatine, types I and IV collagens, while MMP-9 is incapable of direct proteolysis of collagen I and digests collagen type IV. MMP-2 and -9 are both important regulators of vascular and uterine remodeling in a healthy pregnancy. Alterations in the collagen structure of the uterus and spiral arteries are observed in women with hypertensive disorders of pregnancy. Dysregulation of MMP-2 and MMP-9 has been implicated in abnormal vasodilation, placentation, and uterine expansion in preeclampsia. Early preeclampsia detection is paramount for risk stratification and prevention of further complications. Understanding the role of MMP-2 and-9 in uteroplacental and vascular remodeling could help design new approaches for prediction and management of preeclampsia. This review presents a general survey of MMP-2 and MMP-9 faulty regulation and impaired collagen types I and IV turnover in complicated pregnancies. Their potential role as circulating markers for diagnosis, prognosis, and monitoring of preeclampsia development is discussed as well.

## 1. Introduction

Preeclampsia (PE) is one of the most significant causes of maternal and perinatal morbidity and mortality. It has been estimated that around 2–8 per cent of pregnancies in the world are complicated by PE. Fetal growth restriction is also generally seen. The mechanisms of PE have not yet been clearly identified. There is evidence that vascular resistance is abnormally increased and leads to hypertension in women with PE, but the causal mechanisms leading to preeclampsia have not yet been fully estimated. Poor placental perfusion plays an important role in increasing maternal vascular resistance, thus, it has been proposed as a significant reason for subsequent placental ischemia. Impaired expression of matrix metalloproteinases has been associated with shallowing cytotrophoblast invasion and incomplete remodeling of the spiral arteries. Studies have demonstrated placental ischemia and hypoxia as a central causative factor in the etiology of preeclampsia. Hence, altered placental perfusion has been highlighted as a major reason leading to widespread maternal endothelial dysfunction.

## 2. General Concepts of Extracellular Matrix of Uterus and Vasculature

A normal pregnancy is associated with marked hemodynamic and uterine changes that allow adequate uteroplacental blood flow and uterine expansion for a growing fetus. It has been reported that extracellular matrix (ECM) might modulate trophoblast invasion and play a key role in the remodeling of the decidua at the maternal fetal interface. There is evidence that during a healthy pregnancy abundant placentation and extensive remodeling of the spiral arteries in the decidua basalis occur. The extravillous trophoblasts invade the decidua and extend into the walls of the spiral arteries, replacing the endothelium and muscular wall, and create dilated low-resistance vessels that maintain an adequate blood and nutrient supply to the developing fetus. These changes associated with pregnancy implicate significant remodeling of uteroplacental and vascular tissue. Therefore, it can be concluded that the extracellular matrix of uterus, placenta, and vasculature breaks down and remodels during physiological pregnancy.

## 3. Type I and Type IV Collagen Characteristics

Type I collagen (COL1) is an essential connective tissue protein. Its major function is to increase the cytoskeleton’s strength and stability. A long protein chain, characterized by repetitive amino acid residues and a normal secondary structure, is involved in the extraordinary strength of the skin, ligaments, tendons, and vessels. Type I collagen is collected in fibers, thus, forming a structural-mechanical framework. “The dominant isoform of COL1 is represented by heterotrimers of two alpha1 (I) and one alpha2 (I) chains”. The homotrimeric isoform is known to be more cleavage resistant than collagenases. This may clarify its unusual accumulation and its essential function in tumor and fibrosis pathogenesis [1].

Collagen I is fibrillar collagen and a major part of the interstitial membrane’s structure. COL1 is the most common type of collagen and a fundamental component in the structure of many tissues. Practically, it is present in all body systems containing connective tissue. Collagen I is a major structural protein of bone, skin, tendon, ligaments, sclera, cornea, and blood vessels, as well as an essential component of uterus. Human uterus is composed of a fibrous tissue framework that consists mainly of collagen types I and III. They both are responsible for the coherence and supportive strength of the uterus [1].

Type I procollagen is formed by a combination of two pro-alpha1 (I) chains with a pro-alpha2 (I) chain. Enzymes outside the cell process these triple-stranded procollagen molecules. After processing, these molecules assemble themselves into long, thin fibrils that cross link to each other in the spaces around cells. The cross-links result in the formation of solid mature type I collagen fibers [2,3]. COL1 provides tensile tendons and fascia’s tensile stiffness and gives bone its biomechanical properties [4].

Type IV collagen (COL4) is a major structural component of the basement membrane has the role of a framework [5]. “COL4 is the main basement membranes’ structural scaffold and exists as an open three-dimensional network that is ideally suited for the close integration of laminin and heparan sulfates” [6,7]. Type IV collagen consists of three long triple-helical collagen α-chains. In every third position of the collagenous domain, glycine residues are presented. The COL4 collagenous domain includes some interruptions in the Gly-X-Y-repeat sequence, unlike rod-like fibrillary collagens, making the molecule more versatile [8].

Type IV collagen is uniquely present in basement membranes of spiral arteries (most distal part of uterine arterial vasculature) and represents their predominant structural element. In a normal pregnancy, uterine spiral arteries undergo physiological structural and functional changes in order to supply the fetus with blood and nutrients.

## 4. General Features of Matrix Metalloproteinases (MMPs)

The MMPs are a group of more than 25 Zn^2+^-requiring endopeptidases with overlapping activities against a variety of ECM components [9,10]. They are proteolytic enzymes with common functional domains and an action mechanism associated with the degradation of ECM components. MMPs are proteases that are zinc dependent and they can be activated by various types of cytokines and growth factors [11]. One of the most decisive properties of metalloproteinases is their ability to bind to components of the extracellular matrix and, in particular, to collagen and elastin. Activated macrophages in the wall of arterial vessels secrete MMPs. From the perspective of normal and pathological physiology, the capacity of MMPs to change tissues is crucial. On the basis of the current data, MMPs are classified into groups according to the type of proteolytic substrate (extracellular matrix component) against which they act and degrade, respectively. These groups are as follows: collagenases (MMP-1 and MMP-13), gelatinases (MMP-2 and MMP-9), stromelysins such as MMP-3, and membrane MMPs [12].

## 5. Characteristics of Matrix Metalloproteinases-2 and -9

MMP-2 and MMP-9 play a central role in endometrial tissue remodeling during a healthy pregnancy, because they are able to degrade components of the ECM. MMP-2 and MMP-9 are frequently implicated as important factors that contribute to the cytotrophoblast invasion into the maternal vasculature. This assumes that MMP-2 and MMP-9 are implicated in the remodeling of placental and uterine artery. It has been postulated that MMP-2 and MMP-9 play a crucial role during pregnancy, being involved in the degradation of collagen I and IV, thus, implementing a central part in endometrial tissue remodeling during pregnancy. Therefore, MMP-2 and MMP-9 are regarded to be the key enzymes during implantation. The control of expression and regulation of MMPs and their inhibitors plays an important role in healthy and complicated pregnancies. There is increasing evidence that dysregulation of MMP-2 and -9 has been associated with PE.

Gelatinases (MMP-2 and MMP-9) easily digest gelatin. This process is favored by their three fibronectin type II repeats that bind to gelatin/collagen. Different ECM molecules are also digested, including types IV, V, and XI collagens; laminin; aggrecan core protein; etc. Similar to collagenases, MMP-2, but not MMP-9, digests collagens I, II, and III [13,14]. The collagenolytic activity of MMP-2 in solution is much weaker than MMP-1, however, since “proMMP-2 is recruited to the cell surface and activated by the membrane-bound MT-MMPs, it may express reasonable collagenolytic activity on or near the cell surface” [15].

### 5.1. MMP-2 Structure and Function

MMP-2 (gelatinase A, type IV collagenase) is one of the two discovered human gelatinases that are named for their ability to degrade gelatin (denatured collagen) proteolytically. “MMP-2 is ubiquitously expressed as a 72-kDa proenzyme and subject to extensive glycosylation” [16]. In contrast to MMP-9, MMP-2 expression is constitutive and most proinflammatory stimuli fail to raise the degree of expression, because the gene lacks binding sites for proinflammatory transcription factors such as activator protein-1 [17]. The activation status of MMP-2 is crucial for its function in angiogenesis.

The catalytic domain, containing cysteine-rich inserts resembling collagen-binding regions of the type II repeats in fibronectin, is a feature that distinguishes MMP-2 from other MMPs. Binding and cleavage of collagen and elastin need these inserts [18]. MMP-2 is capable of cleaving gelatin; types I, IV, and V collagens; elastin; and vitronectin [19]. Gelatinases degrade collagen in the vascular basal membranes. MMP-2 can also facilitate cell migration by direct degradation of the basement membrane allowing neutrophils and lymphocytes to infiltrate, or releasing chemoattractants, for example, [20]. MMP-2 is involved in both activating and inhibiting inflammation by releasing proinflammatory mediators, such as the active form of interleukin-1β and proteolytic degradation of chemoattractants.

### 5.2. MMP-9 Structure and Function

MMP-9 (gelatinase B, type IV collagenase) was firstly detected in neutrophils in 1974 [21]. “MMP-9 is expressed in the form of 92-kDa proenzyme, which can be activated to the 83-kDa mature enzyme” [22]. Noteworthy, the activation of MMP-9 can be mediated by removal of the prodomain by serine proteases or other MMPs [23]. An alternative pathway can be a direct response to oxidative stress that disrupts the cysteine switch [24].

MMP-9 is incapable of direct proteolysis of collagen I [19]. Collagen types IV, V, VII, X, and XIV; fibronectin; and laminin have been reported to be digested by MMP-9 [25]. The biologically active form of vascular endothelial growth factor (VEGF) is released by MMP-9, thus, playing a key role in angiogenesis. The direct proteolytic degradation of vascular basement membrane proteins complements this process, demonstrating that MMP-9 (even more than MMP-2) could play an important role in the formation of new blood vessels. It has been found that in a xenograft model, the hemopexin domain of MMP-9 was an inhibiting factor in angiogenesis, as demonstrated by decreased invasion of glioblastoma cells overexpressing the MMP-9 hemopexin domain [26].

MMP-9 plays important roles in physiological processes such as reproduction, growth, and development [27,28]. The functions of MMP-9 in angiogenesis are exerted in physiological, as well as pathological conditions, for example, premature rupture of membranes [29,30,31]. MMP-9 is able to process cytokines and chemokines similar to MMP-2. It has been postulated that MMP-9 cleaves interleukin-8 to its more potent truncated form, activates IL-1b, and transforms growth factor beta [19]. MMP-2 is known to be mainly inhibited by TIMP-2, while MMP-9 is primarily inhibited by TIMP-1 [32]. Universally, MMP-2 is expressed under physiological conditions, while MMP-9 is present only constitutively in neutrophils, where it is stored in granules to be released quickly after stimulation [33]. In many other cell types, the expression is inducible by (inflammatory) stimuli [34], increased malignant cell lines, and correlates with their metastatic potential [35]. As previously mentioned, MMP-9 was first discovered in neutrophils, whereas proMMP9 is TIMP-free in neutrophils [21]. Neutrophil-derived MMP-9 is distinguishable from other sources, since it forms a covalent complex with neutrophil gelatinase B-associated lipocalin (NGAL) [36].

Collagen is the main structural component of connective tissue. Matrix metalloproteinases are responsible for breaking down collagen and other extracellular matrix proteins. In physiological conditions, MMPs are released into the extracellular space, thus, degrading the ECM and favoring tissue remodeling and repair. MMP-2 and MMP-9 are known to cleave various other substrates other than ECM proteins and they have multiple unique substrates, but the focus of our review is on MMP-2 and -9 being ECM degraders.

## 6. Types I and IV Collagen Turnover in Healthy Pregnancy

Normal placental vascularization is a cornerstone of a healthy pregnancy. Uterine spiral arteries transform physiologically to ensure sufficient blood and nutrient supply to the fetus. The spiral arteries’ main function is to supply the placenta. Hereby, in a healthy pregnancy, they are modified for uteroplacental blood flow. This process involves loss of smooth muscle and the elastic lamina from the vessel wall as far as the inner third of the myometrium and is associated with a five- to ten-fold dilation at the vessel mouth. During early pregnancy, the placenta plays an important role as a maternal-fetal interface through several processes including vasculogenesis, angiogenesis, trophoblast invasion, and vascular remodeling [37]. Failure of the physiological conversion of the spiral arteries can cause a number of complications, including intrauterine growth restriction and preeclampsia [38]. MMPs and their inhibitors are known to play a crucial role in trophoblast invasion into the uterine wall [39]. MMPs play an important function in the profound changes involving the microarchitecture of the uterus. These changes are required for spiral vessels’ transformation and create an optimum environment for embryonic development, thus, involving a grounding transformation [40]. The blastocyst is known to attach to the uterine wall. This process leads to a complex dialogue between membrane ligands and receptors that penetrate the epithelium and cross the basal lamina [41]. The invasion of trophoblast cell is strictly regulated by signaling events, autocrine and paracrine stimulus, specific protein recognition, and immunological tolerance, promoting MMPs and inhibiting factors TIMPs [42]. It can be accented that MMP-2 and -9 are associated with ECM remodeling and trophoblast invasion of the spiral arteries in both healthy and complicated pregnancy.

The major structural proteins in the uterine wall have been found to be collagen types I and III. When the uterus expands during a pregnancy, collagen turnover intensifies. The connective tissue proteins COL1 and COL4 are essential for structural stabilization and control of cell growth and differentiation. Collagen types I and III synthesis and degradation in the human uterine ECM are dynamic processes that reflect a healthy and complicated pregnancy. In order to provide expansion of the growing uterine content, regulated collagenolysis and/or changes in collagen cross-linking is required. Type IV collagen is uniquely present in basement membranes of spiral arteries (the most distal part of uterine arterial vasculature) and represents their predominant structural element [43,44]. In a healthy pregnancy, uterine spiral arteries undergo physiological structural and functional changes to supply the fetus with blood and nutrients.

As previously mentioned, MMP-2 and MMP-9 are important regulators of uterine and vascular remodeling. A normal pregnancy is associated with extensive uterine and spiral arteries remodeling and proteolysis of extracellular matrix, mediated by matrix metalloproteinases (MMPs) [45]. Furthermore, remodeling of cervical tissue is known to be significant, with decreased concentrations of collagen and proteoglycans concomitant with increased collagenolytic activity [46,47].

## 7. Impaired Types I and IV Collagen Turnover in Preeclampsia: The Role of MMP-2 and -9 Dysregulation

Matrix metalloproteinases are known to play important role in modulation of vascular and uterine remodeling. Studies have indicated that increases in MMP-2 and MMP-9 are implicated in vasodilation, placentation, and uterine expansion during a normal pregnancy. Of note, decreased vascular MMP-2 and MMP-9 may lead to decreased vasodilation, increased vasoconstriction, hypertensive disorders of pregnancy, and preeclampsia. MMP expression/activity are thought to be impaired during complications of a pregnancy. This might be a reason for shallow cytotrophoblast invasion and incomplete remodeling of the spiral arteries. According to the current approaches, MMPs may promote vasoconstriction and surface receptor cleavage affecting the vasculature. Hence, MMP-2 and -9 are suggested to have a key regulatory role in developing placental ischemia which favors preeclampsia.

Whether the uteroplacental and vascular MMP balance is altered in hypertensive disorders of pregnancy (HDP) is a topic of interest [48]. How MMP imbalance could affect uteroplacental remodeling and vascular function are emerging areas for new research. The altered MMP-2 and -9 expression/activity in preeclampsia might be a cause for abnormal uteroplacental and vascular remodeling. This is associated with impaired trophoblast invasion [49,50] and results in placental ischemia. Abnormal placentation, reduction of uterine perfusion pressure (RUPP), and placental ischemia play important roles in the development of preeclampsia [51,52,53]. Collagen structure has been shown to be disturbed in women with preeclampsia [44].

During preeclampsia, the uterine extracellular matrix metabolism has been found to be altered. Although extensive researcher efforts have been conducted on this problem, little is known about MMP-2 and-9 dysfunction in PE. A topic of great interest is the mechanism inducing changes in the composition of the collagen in the human uterus and spiral arteries. Although the pathogenesis of preeclampsia is not well understood, inadequate placental perfusion is recognized as a significant pathway contributing to widespread maternal endothelial dysfunction. Impaired placental expression of matrix metalloproteinases has been theorized to shallow cytotrophoblast invasion and incomplete remodeling of the spiral arteries. It is also assumed that MMPs might favor vasoconstriction, thus, relating placental ischemia to preeclampsia’s cardiovascular alterations [54,55].

**Figure 1:**
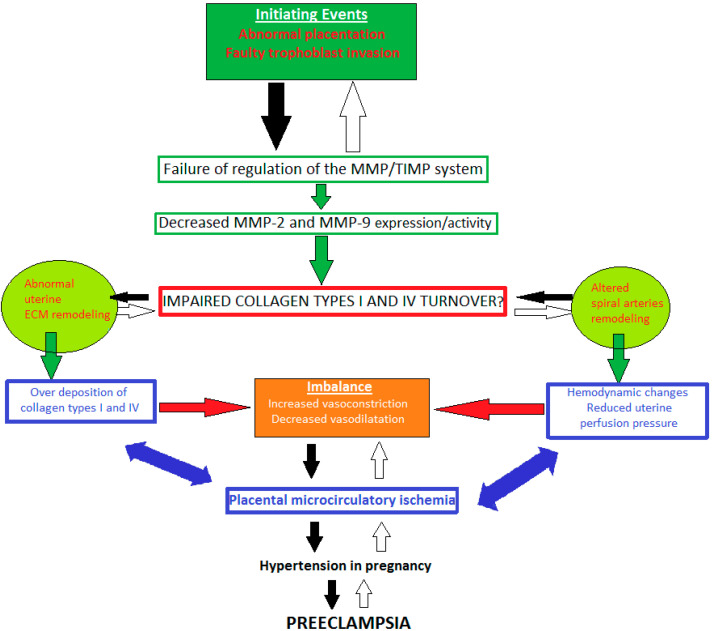
A possible schematic pattern illustrating the eventual mechanisms of impaired collagen I and IV turnover in pathological pregnancy.

A widely held concept is that initial faulty trophoblast invasion and abnormal placentation are considered to be initiating events [39]. Thus, the degradation activity of gelatinases MMP-2, and -9 is disturbed. Decreased MMP-2 and -9 expression/activity could lead to impaired collagen types I and IV turnover, which might affect the remodeling of uterine ECM and spiral arteries [56,57]. Various cytoactive factors are released triggering an incessant cycle of over deposition of collagen, suppressed trophoblast invasion of spiral arteries, angiogenic imbalance (presented by increased vasoconstriction and reduced vasodilation), generalized RUPP, and disturbed blood flow to both the placenta and fetus. All these processes might be reason for uteroplacental ischemia. Therefore, a vicious cycle is activated and suppressed trophoblast invasion of spiral arteries and RUPP deteriorate. Placental ischemia progresses, causing hypertension in pregnancy, preeclampsia, and intrauterine growth restriction; however, the molecular mechanisms and the abnormal regulation of MMP-2 and -9 expression/activity contributing to the genesis and progression of hypertensive disorders of pregnancy are unclear [39,56,58,59] (Figure 1).

The investigation of Karthikeyan et al. [60] highlighted that failure of the MMP/TIMP system (through plasma and genetic alterations) for controlling the extracellular matrix remodeling may lead to gestational hypertension and preeclampsia. Furthermore, vascular remodeling disorders of the uterine and placenta hypoperfusion have been generally recognized in prior years [61]. A number of studies have evaluated the key role of matrix metalloproteinase-2 and -9 in healthy [62,63,64,65] and complicated pregnancies [66,67,68,69,70,71,72,73,74,75,76,77,78,79,80,81,82,83,84].

### 7.1. MMP-2 Dysregulation

In an important investigation, Li et al., 2014, examined maternal and fetal parameters and MMPs expression, activity, and distribution associated with hypertensive disorders in pregnancy. They measured collagen and elastin content in the uterus, placenta, and aorta of normal pregnancy rats and in rat RUPP model. The results indicated that maternal blood pressure was higher; uterine, placental, and aortic weight and the litter size and pup weight were less in RUPP than normal pregnancy rats. The gelatin zymography and Western blots both demonstrated decreases in amount and gelatinase activity of MMP-2 and MMP-9 in the uterus, placenta, and aorta of RUPP as compared with normal pregnancy rats. Of note, immunohistochemistry confirmed reduced MMPs in the uterus, placenta, and aortic media of RUPP rats [66].

Ren et al., 2018, tested the hypothesis that placental ischemia-induced changes in soluble fms-like tyrosine kinase-1 (sFlt-1) and placental growth factor (PlGF) target vascular and uteroplacental matrix metalloproteinases (MMPs). Their study showed that if soluble sFlt-1/PlGF imbalance was corrected by infusing PlGF, decreases in vascular and uteroplacental MMP-2 and MMP-9 could be reversed, as well as increases in MMP-1, MMP-7, and collagen types I and IV induced by placental ischemia and antiangiogenic sFlt-1 could be reversed in hypertensive disorders in pregnancy. Interestingly, changes in vascular and uteroplacental MMPs and collagen content might be regulated by angiogenic factors and MMP modulators. Hence, hypertension and intrauterine growth restriction ameliorate in preeclampsia [67].

Lin et al., 2020, used an experimental model of rats with hypertension in pregnancy. Their studies showed decreased number, size, and expansiveness of uterine spiral arteries. Moreover, MMP-2 and MMP-9 were decreased, while collagen IV was increased. The authors concluded that the decreased uterine vascularization and uterine arterial expansive remodeling by MMPs could contribute to progressive uteroplacental ischemia in hypertension in pregnancy and preeclampsia [56,68].

In order to evaluate whether dimerization of matrix metalloproteinases plays a role in hypertensive pregnancy (HTN-Preg) and intrauterine growth restriction (IUGR) Chen et al., 2017, measured the levels/activity of MMP-9, tissue inhibitor of metalloproteinase (TIMP-1), and their dimerization forms in the placenta, uterus, and uterine artery of normal pregnant rats and a rat model of RUPP. They reported that the increased blood pressure and decreased pup weight in placental ischemia model of HTN-Preg were associated with a decrease in MMP-9 homodimer and an increase in MMP-9/TIMP-1 complex in placenta, uterus, and uterine artery. These results indicate a net decrease in MMP-9 activity and reduced uteroplacental and vascular remodeling in the setting of HTN-Preg and IUGR. Chen, J. et al. concluded that enhancing epidermal growth factor receptor/protein kinase C signaling may reverse the MMP-9 unfavorable dimerization patterns, and thereby promote uteroplacental and vascular remodeling in preeclampsia [69].

Dias-Junior et al., 2017, focused on the regional differences in angiogenic balance and matrix metalloproteinases. The investigators examined whether these indicators underlie regional uteroplacental vascularization and feto-placental development. Their study compared fetal and placental growth, and placental and myoendometrial vascularization in the proximal, middle, and distal regions of the uterus (in relation to the iliac bifurcation) in normal pregnant (Preg) and RUPP rats. They concluded that localized angiogenic imbalance and decreased MMP-2 and MMP-9 could cause further decline in placental and myoendometrial vascularization and placental and fetal growth in distal as compared with proximal uterus of HTN-Preg rats, in addition to a general reduction in placental and fetal growth during uteroplacental ischemia. Of note, regional differences in uteroplacental perfusion, angiogenic balance, and MMPs could be a factor in the incidence of preeclampsia in multiple pregnancy [70].

Laskowska in 2017, tested the hypothesis that maternal serum matrix metalloproteinases-2, -3, -9, and -13 levels could differ in early- and late-onset preeclampsia and uncomplicated pregnancies. Maternal serum MMP-2 levels were significantly higher than those in the controls in both the groups of preeclamptic women. MMP-3 levels were significantly higher in patients with early-onset preeclampsia; though the MMP-3 levels in patients with late-onset preeclampsia were similar to those observed in healthy controls. The MMP-9 levels were reported to be lower, whereas the levels of MMP-13 were higher in both preeclamptic groups of pregnant women than in the control subjects, however these differences were not statistically significant. A major conclusion of this study was that higher levels of MMP-2 and MMP-13 and lower levels of MMP-9 are possibly associated with both early- and late-onset severe preeclampsia [71].

The circulating plasma MMP-2 and -9 levels, TIMP-1 and TIMP-2 in women who subsequently develop preeclampsia, were explored by Myers et al., 2005, via zymography and Western blot. The study focused on women whose pregnancies were subsequently complicated by preeclampsia. The investigators took samples from a control group including normal pregnant women at 22 and 26 weeks and at delivery or diagnosis. They reported an imbalance in the MMP-2/TIMP-1 ratio at all three gestational time points in patients who subsequently developed preeclampsia [72].

The cross-sectional study by Palei 2008, included women with preeclampsia, gestational hypertension, normotensive pregnancies, and healthy nonpregnant women. The authors assessed the circulating levels of Pro-MMP-2, Pro-MMP-9, TIMP-1, TIMP-2, and the Pro-MMP-9/TIMP-1 and Pro-MMP-2/TIMP-2 ratios. Gelatin zymography and enzyme linked immunosorbent assay (ELISA) were both applied for measuring pro-MMP and TIMP concentrations in plasma samples. Higher TIMP-1 levels were found in PE as compared with gestational hypertension (GH) and normotensive pregnant women. The TIMP-2 levels in nonpregnant women were lower than those found in normotensive pregnant controls, gestational hypertension, and preeclampsia. The MMP-9 activity in gestational hypertension was increased [73].

Palei 2012, found that pregnant women with preeclampsia had significantly higher plasma MMP-2 and TIMP-2 concentrations than healthy pregnant, although the MMP-2/TIMP-2 ratios were similar. In addition, the plasma MMP-2 levels and MMP-2/TIMP-2 ratios in pregnant women with gestational hypertension were significantly elevated as compared with healthy pregnancies [74].

Martinez-Fierro M. explored MMP-1, -2, -3, -7, -8, -9, -10, -12, and -13 in early-pregnancy urine from 17 women predicted to develop preeclampsia and 48 controls. Interestingly, MMP-2 urinary concentration showed differences between groups. This main finding made possible calculation of an increased risk for PE development of up to 20 times in the study population. The researcher reported that if MMP-2 urinary concentration at 12 and 16 weeks of gestation is increased that might be a predictive indicator for an increased risk of developing preeclampsia in the study population [75].

The main focus of Lavee et al., 2009, was to assess the levels of matrix metalloproteinases and their inhibitors in second trimester amniotic fluid of women with hypertensive disorders and in normotensive women. In order to evaluate their hypothesis, investigators collected amniotic fluid of 133 women undergoing genetic second trimester amniocentesis. MMP was characterized by zymography, whereas MMP-2 levels were measured via ELISA. The authors concluded that higher amniotic fluid MMP-2 and TIMP-2 levels are found in women who eventually develop preeclampsia. [76].

Describing the plasma MMP-2/MMP-9 levels in women with preeclampsia (n = 12) and comparing them to those of women with uncomplicated pregnancies (n = 12) was the major aim of Narumiya et al., 2001. They used zymographic analysis for it. Furthermore, they evaluated the changes in the levels of MMP-2 and MMP-9, as well as tissue inhibitors of MMPs (TIMP-1 and TIMP-2) released by cultured human umbilical vein endothelial cells in response to VEGF (0.1–10 ng/mL). The authors reported that plasma MMP-2 levels were significantly higher in women with preeclampsia as compared with women with uncomplicated pregnancies. The MMP-9 levels were below the level of detection. Moreover, vascular endothelial growth factor (VEGF) stimulated endothelial MMP-2 and MMP-9 release in a concentration- and time-dependent (6–24 h) manner. Of note, VEGF stimulation of MMP release occurred without significantly affecting the release of TIMP-1 and TIMP-2. These findings demonstrate the possibility that VEGF promotes secretion of MMPs from endothelial cells, which, in turn, could alter vascular function in women with preeclampsia [77]. Table 1 summarizes the available literature data for studies measuring levels of MMP-2 in samples of patients with hypertensive disorders of pregnancy.

### 7.2. MMP-9 Dysregulation

Luizon et al., 2014, investigated MMP-9, TIMP-1 polymorphism, plasma TIMP-1 levels, and antihypertensive therapy responsiveness in hypertensive disorders of pregnancy. The levels of plasma MMP-9 and TIMP-1 were measured by ELISA. There were lower MMP-9 levels and MMP-9/TIMP-1 ratios in gestational hypertension patients with the GG genotype for the TIMP-1 polymorphism than in those with the TT genotype. Preeclampsia TG genotype patients had elevated levels of TIMP-1 [78].

Tayebjee et al., 2005, used the ELISA method to evaluate plasma MMP-9 and TIMP-1 and -2 levels in women with gestational hypertension, normotensive women with normal pregnancies, and healthy nonpregnant control subjects. Significant differences were estimated between the three groups regarding circulating MMP-9, TIMP-1 and TIMP-2, and the MMP-9/TIMP-1 and MMP-9/TIMP-2 ratios. Plasma TIMP-1 in non-pregnant subjects was significantly higher than both normotensive pregnant and gestational hypertension subjects. Plasma TIMP-2 in non-pregnant subjects was higher than normotensive pregnant subjects, but significantly lower than gestational hypertension patients. Authors demonstrated altered MMP/TIMP ratios in maternal blood during gestational hypertension. In light of the vascular and cardiac ECM structural changes in hypertension, the investigators suggested that these findings may be related to the pathophysiology of human gestational hypertension [79].

A case-control study by Ab Hamid et al., 2012, involved women with gestational hypertension and normotensive pregnant women. Total levels of MMP-9 and TIMP-1 and -2 were explored. These biomarkers were measured via ELISA. The results indicated low levels of expression of TIMP-1 and TIMP-2 in the gestational hypertension group. The authors reported weak positive correlations between maternal age and TIMP-1 in the gestational hypertension group and between gestational age and TIMP-2 in the control group [80].

In 2014, forty-seven biomarkers involved in the pathogenesis of preeclampsia were investigated in 5623 women by a large analysis called Early Pregnancy Prediction of Preeclampsia in Nulliparous Women, Combining Clinical Risk and Biomarkers, The Screening for Pregnancy Endpoints (SCOPE) International Cohort Study. The conception of study was to measure these biomarkers in plasma sampled at 14 to 16 weeks’ gestation. Decreased MMP-9 levels were reported [81].

The study by Montagnana et al., 2009, investigated MMP-2 and -9 and their inhibitors TIMP-1 and -2 in preeclamptic as compared with normotensive pregnant and non-pregnant women via ELISA [49].

In a systematic expression analysis, Wang et al., 2010, found significantly higher expression levels of MMP-9 in placental sections from preeclampsia tissue and this increased expression was well correlated to promoter demethylation. The investigators demonstrated that the percentage of unmethylated-712 sites were higher in preeclampsia patients as compared with controls. This study demonstrates that altered synthesis of MMP-9 in preeclampsia placentas may result from epigenetic changes of the methylation status of CpG sites in the promoter region [82].

Meng et al., 2016, investigated the expression changes of MMP-9 in serum and placenta tissues of patients with early-onset preeclampsia (EOPE), to explore the relation between MMP-9 and EOPE. ELISA, immunohistochemical staining, and Western blot were used to examine the expression levels of MMP-9 in serum and placenta tissues of both an EOEP group and a control group. The serum level of MMP-9 in the EOPE group was significantly higher as compared with the control group, whereas the MMP-9 expression in placenta tissues was significantly decreased. This study provides evidence that the elevation of serum MMP-9 level and decline of placenta MMP-9 level may be related to the occurrence of EOPE, indicating MMP-9 may be involved in pathological and physiological process of preeclampsia [83].

The expression and characteristics of MMP-9 and TIMP-1 in placenta of pregnancy induced hypertension (PIH) in Uygur women were examined by Zhang, Y. et al., 2019. Their main focus was to analyze the correlation of MMP-9 and TIMP-1 with PIH and provide a theoretical basis for clinical work. The investigation involved 90 cases of placental tissue patients. They were divided into three groups as follows: 30 cases of severe preeclampsia, 30 cases of mild preeclampsia, and 30 cases as a normal group. A statistically significant difference of MMP-9 in the three groups was reported. The results also indicated a statistically significant difference between the severe group and normal and mild groups. Interestingly, with the aggravation of PIH, the positive expression of MMP-9 was gradually decreased. These data provide evidence for two main conclusions. Firstly, the positive expression ratio of MMP-9/TIMP-1 in the severe group was lower than that in normal pregnancy and mild groups. Secondly, the positive expression ratio of the two groups became smaller as the condition worsened. This study also demonstrated that the association between the positive expression of MMP-9 in placental tissue of patients with PIH decreased significantly and the severity of PIH. The authors found that the positive expression of MMP-9 in placental tissue of patients with PIH decreased significantly with the severity of PIH [84]. Table 2 summarizes the available literature data for studies measuring levels of MMP-9 in samples of patients with hypertensive disorders of pregnancy.

## 8. Limitations and Future Prospectus on the Application of MMP-2 and -9 as Preeclampsia Biomarkers

Previous studies have described the measurement of matrix metalloproteinase-2 in samples of experimental animal model involving RUPP rats with hypertensive disorders of pregnancy vs. normal pregnant rats. The data showed decreased levels/activity of MMP-2 [66,67,68,69,70]. Contrary to that, human studies of women with HDP report increased MMP-2 levels/activity [49,71,72,73,74,75,76,77]. Regarding MMP-9, studies have found decreased levels/activity in RUPP rats with HDP [66,67,68,69,70]. Similarly, most human studies involving women with HDP have shown decreased MMP-9 levels/activity [49,71,78,79,81,84], while, in contrast, few investigations have reported increased MMP-9 levels/activity [73,74,80,82,83]. One of the possible explanations could be the fact that serum MMPs may represent the global MMPs production from various maternal tissues such as the uterus and placenta.

When the data on human research is interpreted, it is notable that different studies focus on various patient groups including women with a wide range of hypertensive disorders of pregnancy such as patients with gestational hypertension [79,80], preeclampsia [49,71,72,76,77,81,82,83,84], and both GH and PE [73,74,78]. This makes comparing the results of studies very difficult.

An analysis of all the studies, in this review, indicates that some important differences can be observed between the methods used for the determination of MMP-2 and -9. It appears that researchers have applied a wide range of methods for MMP-2 and -9 detection. For example, zymography was used for determining biomolecules’ activity [66,67,68,69,70,72,77] and ELISA was applied for measuring biomarkers’ concentrations [49,71,76,78,79,80]; both zymography and ELISA [73,74,83], immunohistochemistry [68,84], methylation-sensitive restriction enzymes, and polymerase chain reaction amplification were implemented [82]. A reviewing of the human studies in this article, indicates some differences can be noted between the type of samples used for MMP-2 and -9 detection. Investigations [49,71,80,83] applied serum specimen, while [72,73,74,77,78,79,81] used plasma. Furthermore, [75] implemented urine samples, [76] focused on amniotic fluid, while [82,83,84] involved placenta. These concerns may be of significant importance in the explanation of different studies’ results.

Another significant feature that needs to be pointed out is that most investigations were cross-sectional, and they reported levels of MMP-2 and MMP-9 only at one certain point. Probably, studies estimating these markers by serial measurements at various time points would be more accurate. Moreover, it has not been completely established whether a serum biomarker is fully informative for the tissue level concentration. A possible tool would be a method to associate biomarkers with uterine and spiral arteries remodeling, involving immunohistochemistry and histology of uterine biopsies, but it cannot be performed in cross-sectional studies.

## 9. Conclusions

Dysregulation interplay between MMP-2, -9, and their natural tissue inhibitors TIMP-2 and -1 might be one of the possible causes for pathological collagen types I and IV turnover in preeclampsia. The disturbed metabolism of the abovementioned fibrillar connective tissue proteins could partially explain the uterine ECM changes, manifested as altered structure of uterus and abnormal spiral arteries remodeling in women with preeclampsia. In conclusion, matrix metalloproteinases -2 and -9 have promising potential as biomarkers and might be useful in the diagnosis, prognosis, and monitoring of the development of preeclampsia.

## Figures and Tables

**Table 1:** Levels of MMP-2 in samples of patients with hypertensive disorders of pregnancy.

Authors	Hypertensive Disorder	Sample	Method	Findings
Li et al., 2014 [66]	Normal pregnant vs. RUPP rats	Uterus, placenta, aorta	Zymography	Decreased MMP-2 activity
Ren et al., 2018 [67]	Normal pregnant vs. RUPP rats	Uterus, placenta, aorta	Zymography	Decreased MMP-2 activity
Lin et al., 2020 [56]	Normal pregnant vs. RUPP rats	Uterus, uterine spiral arteries	Zymography Immunohistochemistry Uterine histology	Decreased MMP-2 activity/expression and concentration
Chen et al., 2017 [69]	Normal pregnant vs. RUPP rats	Uterus, placenta, uterine artery	Zymography	Decreased MMP-2 activity
Dias-Junior et al., 2017 [70]	Normal pregnant vs. RUPP rats	Uterus	Zymography	Decreased MMP-2 activity/levels
Laskowska, 2017 [71]	Early onset PE vs. Late onset PE	Serum	ELISA	Increased MMP-2 levels
Myers Jenny et al., 2005 [72]	PE	Plasma	Zymography	Increased MMP-2 activity at 22 and at diagnosis, no difference at 26 week
Palei et al., 2008 and 2012 [73,74]	PE/GH	Plasma	Zymography ELISA	Increased MMP-2 and MMP-2/TIMP-2 ratio in GH
Montegranna et al., 2009 [49]	PE	Serum	ELISA	Increased MMP-2 levels in PE vs. both non-pregnant and physiologic pregnant women
Martinez-Fierro et al., 2018 [75]	Early-pregnancy	Urine	Quantitive evaluation	Increased MMP-2 levels
Lavee et al., 2009 [76]	PE	Amniotic fluid	ELISA	Increased MMP-2 levels in amniotic fluid
Narumiya et al., 2001 [77]	PE	Plasma	Zymography	Increased MMP-2 activity

RUPP, reduced uteroplacental perfusion pressure; MMP-2, matrix metalloproteinase 2; HDP, hypertensive disorders of pregnancy; PE, preeclampsia; GH, gestational hypertension; ELISA, enzyme linked immunosorbent assay.

**Table 2:** Levels of MMP-9 in samples of patients with hypertensive disorders of pregnancy.

Authors	Hypertensive Disorder	Sample	Method	Findings
Laskowska, 2017 [71]	Early onset PE vs. Late onset PE	Serum	ELISA	Decreased MMP-9 levels
Li et al., 2014 [66]	Normal pregnant vs. RUPP rats	Uterus, placenta, aorta	Zymography	Decreased MMP-9 activity
Ren et al., 2018 [67]	Normal pregnant vs. RUPP rats	Uterus, placenta, aorta	Zymography	Decreased MMP-9 activity
Lin et al., 2020 [56]	Normal pregnant vs. RUPP rats	Uterus, uterine spiral arteries	Zymography Immunohistochemistry Uterine histology	Decreased MMP-9 activity/expression and concentration
Chen et al., 2017 [69]	Normal pregnant vs. RUPP rats	Uterus, placenta, uterine artery	Zymography	Decreased MMP-9 activity
Dias-Junior et al., 2017 [70]	Normal pregnant vs. RUPP rats	Uterus	Zymography	Decreased MMP-9 activity/levels
Luizon et al., 2014 [78]	PE, GH	Plasma	ELISA	Decreased MMP-9 levels andMMP-9/TIMP-1 ratio in GH patients with GG genotype
Taybjee et al., 2004 [79]	GH	Plasma	ELISA	Decreased MMP-9 levels
SCOPE [81]	PE	Plasma		Decreased MMP-9 levels
Montegranna et al., 2009 [49]	PE	Serum	ELISA	Decreased MMP-9 levels in PE vs. normal pregnant women (Non-significantly)
Zhamg et al., 2019 [84]	PE	Placenta	Immunohistochemistry	Decreased MMP-9 expression with aggraviation of pregnancy induced hypertension
Palei et al., 2008, 2012 [73,74]	PE/GH	Plasma	Zymography ELISA	Increased MMP-9 activity in GH
Ab Hamid et al., 2012 [80]	GH	Serum	ELISA	Increased MMP-9 levels
Wang et al., 2010 [82]	PE	Placenta	MSR, PCR	Increased MMP-9 expression levels
Meng et al., 2016 [83]	PE	Placenta, Serum	ELISA Western blot	Increased serum MMP-9 levels Decreased MMP-9 expression in placenta

RUPP, reduced uteroplacental perfusion pressure; MMP, matrix metalloproteinase; HDP, hypertensive disorders of pregnancy; PE, preeclampsia; GH, gestational hypertension; ELISA, enzyme linked immunosorbent assay; MSR, methylation-sensitive restriction enzymes; PCR, polymerase chain reaction amplification.
